# Pituitary Macroadenoma: A Conservative Approach to Apoplexy

**DOI:** 10.7759/cureus.97625

**Published:** 2025-11-24

**Authors:** Celina Gupta, Prethivan Gopalakrishnan, Tala Balafshan

**Affiliations:** 1 Diabetes and Endocrinology, Whiston Hospital, Prescot, GBR

**Keywords:** apoplexy, conservative management, cranial neuropathy, macroadenoma, pituitary, pituitary apoplexy

## Abstract

Pituitary apoplexy represents an acute neuroendocrine emergency that may present with headache and cranial nerve dysfunction. A 54-year-old woman presented with a two-day history of frontal and temporal headache and a painful left surgical third nerve palsy. Initial CT of the head was unremarkable, but MRI revealed an asymmetrically enlarged left pituitary gland with slight suprasellar extension and partial encroachment on the left cavernous sinus, without optic nerve or chiasmal compression. There were features suggestive of apoplexy. Biochemical tests showed normal pituitary hormone levels, except for mildly elevated insulin-like growth factor 1 (IGF-1) and slightly low thyroid-stimulating hormone (TSH), suggesting a slight growth hormone axis dysfunction without clinical acromegaly. While pituitary apoplexy is typically managed surgically, this case highlights successful conservative management with close clinical and radiological monitoring. The patient’s symptoms stabilized without progression, demonstrating that select cases of pituitary apoplexy with cranial nerve involvement may be safely managed nonoperatively.

## Introduction

Pituitary adenomas are benign epithelial tumors arising from the anterior pituitary gland. They are frequently identified incidentally on neuroimaging performed for unrelated reasons [1]. These adenomas are classified based on size into microadenomas (<10 mm) and macroadenomas (≥10 mm). Although the majority are non-functioning [2], they may produce symptoms through mass effect, including headache, visual disturbance, or mild hyperprolactinemia due to stalk compression.

Pituitary apoplexy is a clinical condition that is characterized by rapid hemorrhage or infarction within a pituitary adenoma, resulting in the rapid increase of intrasellar contents, leading to a rise in intrasellar pressure. Tumor vulnerability arises from its high metabolic demand, sparse vascularity, and sensitivity to systemic hemodynamic shifts [3,4].

The clinical presentation reflects the direction of mass effect. As hemorrhage and necrosis progress, compression of the pituitary gland and stalk can precipitate acute hypopituitarism, while extension beyond the sella compromises the optic chiasm, causing bitemporal hemianopia, whereas lateral expansion toward the cavernous sinus can lead to cranial neuropathies. The oculomotor nerve is particularly susceptible due to its superficial location in the sinus wall. MRI is the imaging modality of choice, typically demonstrating T1 hyperintensity from hemorrhage, especially if the MRI is done after a few days [4,5]. These features were evident in this patient, with a high T1 signal and partial cavernous sinus encroachment despite a normal CT scan.

Although surgical decompression is traditionally recommended, especially with progressive visual loss, reduced consciousness, or chiasmal compression, recent evidence supports conservative management in stable patients [4]. This case is unusual in presenting with isolated surgical third cranial nerve palsy and early spontaneous improvement. In the absence of chiasmal involvement or clinical deterioration, the patient was successfully managed nonoperatively, contributing to emerging evidence that selected cases of pituitary apoplexy can be treated conservatively with close multidisciplinary follow-up.

## Case presentation

A 54-year-old patient presented with a two-day history of frontal and temporal headache. She also described a "crushing pain" in the corner of her left eye. She also reported multiple episodes of vomiting. Her past medical history included type 2 diabetes mellitus (T2DM), migraine headaches, and generalized anxiety disorder.

On examination, she was alert and oriented (Glasgow Coma Scale score = 15/15), with stable vital signs. Neurological assessment revealed a left-sided, dilated third cranial nerve palsy, manifesting as ptosis and impaired saccadic movements. Fundoscopy was unremarkable, and there were no true visual defects. The remainder of the cranial nerve and systemic examination was within normal limits.

Diagnostic assessment

**Figure 1:**
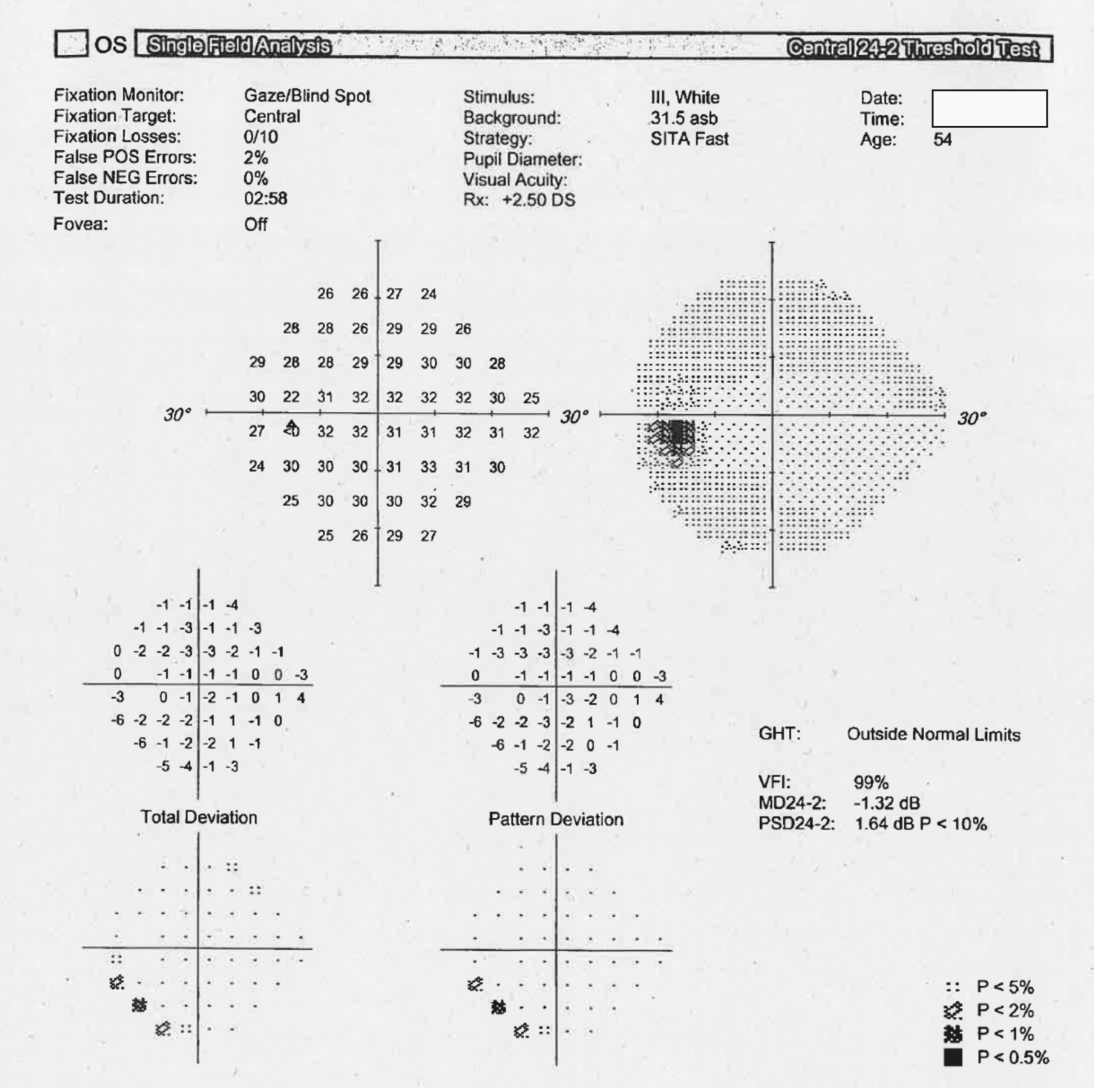
Visual field examination of the left eye. Visual field testing of the left eye (OS) using the Humphrey 24-2 SITA Fast strategy demonstrated good reliability, with no fixation losses and minimal false-positive errors. In particular, there was evidence of a mild inferior temporal field defect on both the total and pattern deviation plots. These findings are consistent with a partial involvement of visual pathways secondary to surgical third nerve palsy and correlate with the clinical finding of left exotropia and right hypertropia.

Neurological examination revealed a sluggish left pupillary response consistent with a painful surgical third nerve palsy. The initial orthoptic assessment showed left eye diplopia, accompanied by headaches and vomiting upon lifting the eyelid. Visual acuity was reduced (with the right eye showing 0.1 and the left eye showing 0.2 on the logMAR chart). The alignment revealed an exotropia (outward turn of the left eye) with a right hypertropia, more pronounced in left gaze and at distance, consistent with surgical third nerve palsy (as shown in Figure 1).** **The right eye was unaffected, and there were no signs of optic disc swelling, diplopia, reduced visual acuity, exotropia, or hypertropia (visual field defects). The calculated Pituitary Apoplexy Score (PAS) was 3/10 (calculated using the scoring system presented in Table 1).

**Table 1:** Pituitary Apoplexy Score (PAS) table. * No change from premorbid visual acuity. Adapted from: Rajasekaran et al. [5]. Reproduced with permission from John Wiley and Sons. License number: 6132080153450.

Variable	Points
Level of consciousness	
Glasgow Coma Scale = 15	0
Glasgow Coma Scale < 8-14	2
Glasgow Coma Scale < 8	4
Visual acuity	
Normal* 6/6	0
Reduced - Unilateral	1
Bilateral	2
Visual field defects	
Normal	0
Unilateral defect	1
Bilateral defect	2
Ocular paresis	
Absent	0
Present - Unilateral	1
Bilateral	2

**Figure 2:**
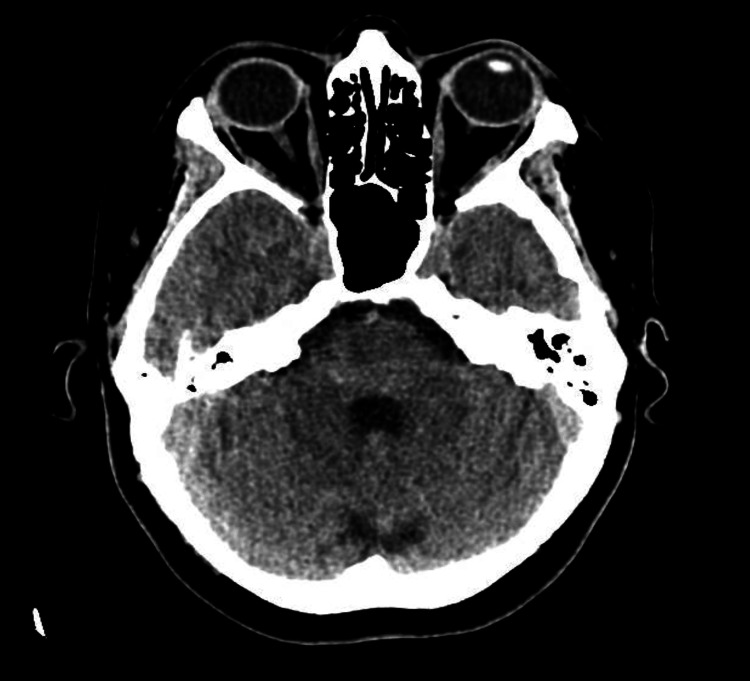
CT of the head. The ventricles and basal systems were patent, allowing for age-related cortical evolution with no disproportionate low atrophy. No acute intra- or extra-axial hemorrhage, large vessel territorial infarct, or parenchymal mass lesion was identified within the limitations of this non-contrast enhanced examination. No acute skull or base fracture was noted, with benign hyperostosis frontalis interna.

**Figure 3:**
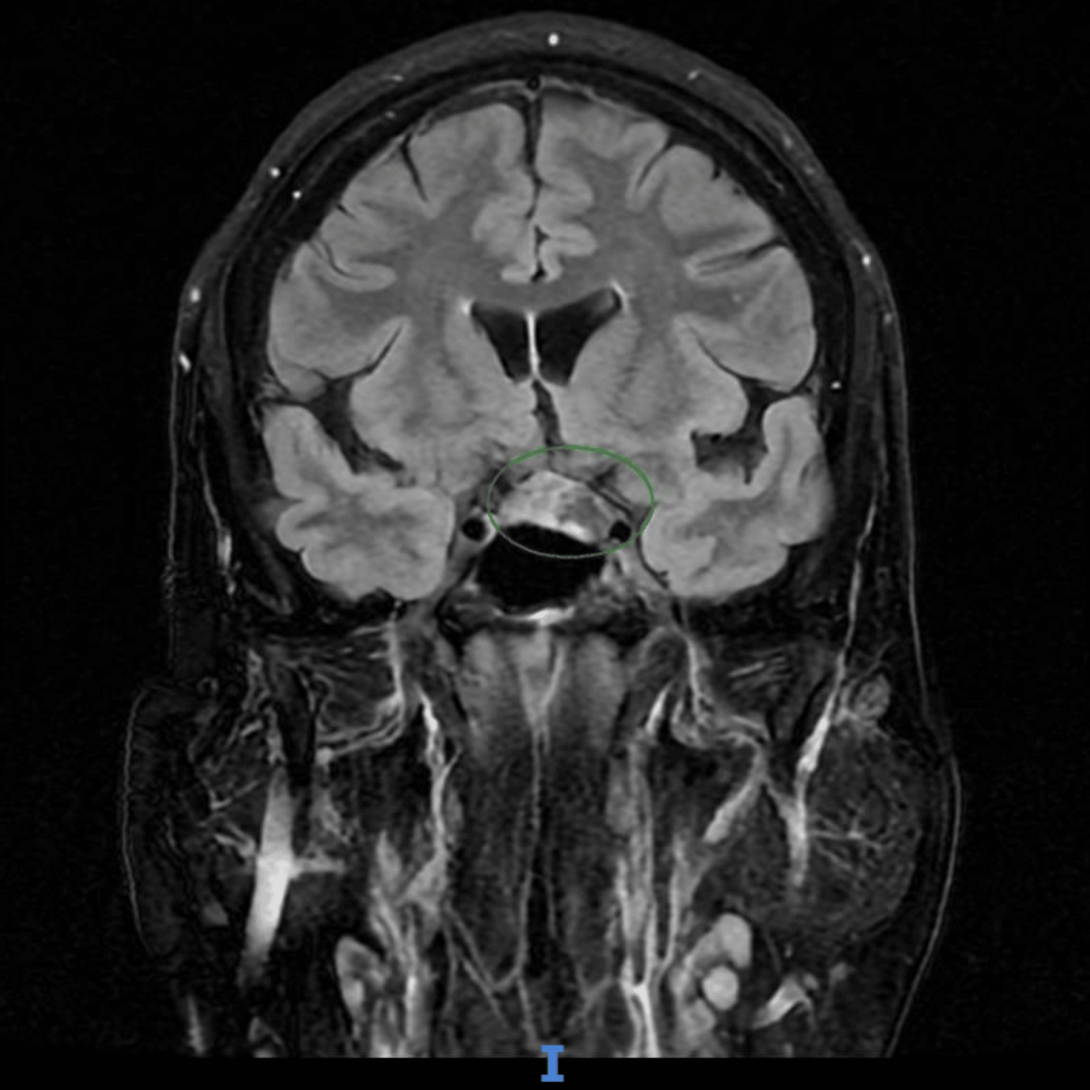
MRI of the head. There was a bulky/enlarged left pituitary gland, with high T1-weighted (T1W) signal, and corresponding low T2 signal, in the left lobe, which may represent hemorrhagic changes. A 0.4 cm non-specific focus of high T2-weighted (T2W)/flare signal in the left cerebral hemisphere - corona radiata was noted. A single focus of susceptibility artefact in the left per occipital deep white matter on susceptibility weighted imaging was present. Otherwise, the normal appearance of the brain parenchyma and ventricles was present, with no acute infarct.

**Figure 4:**
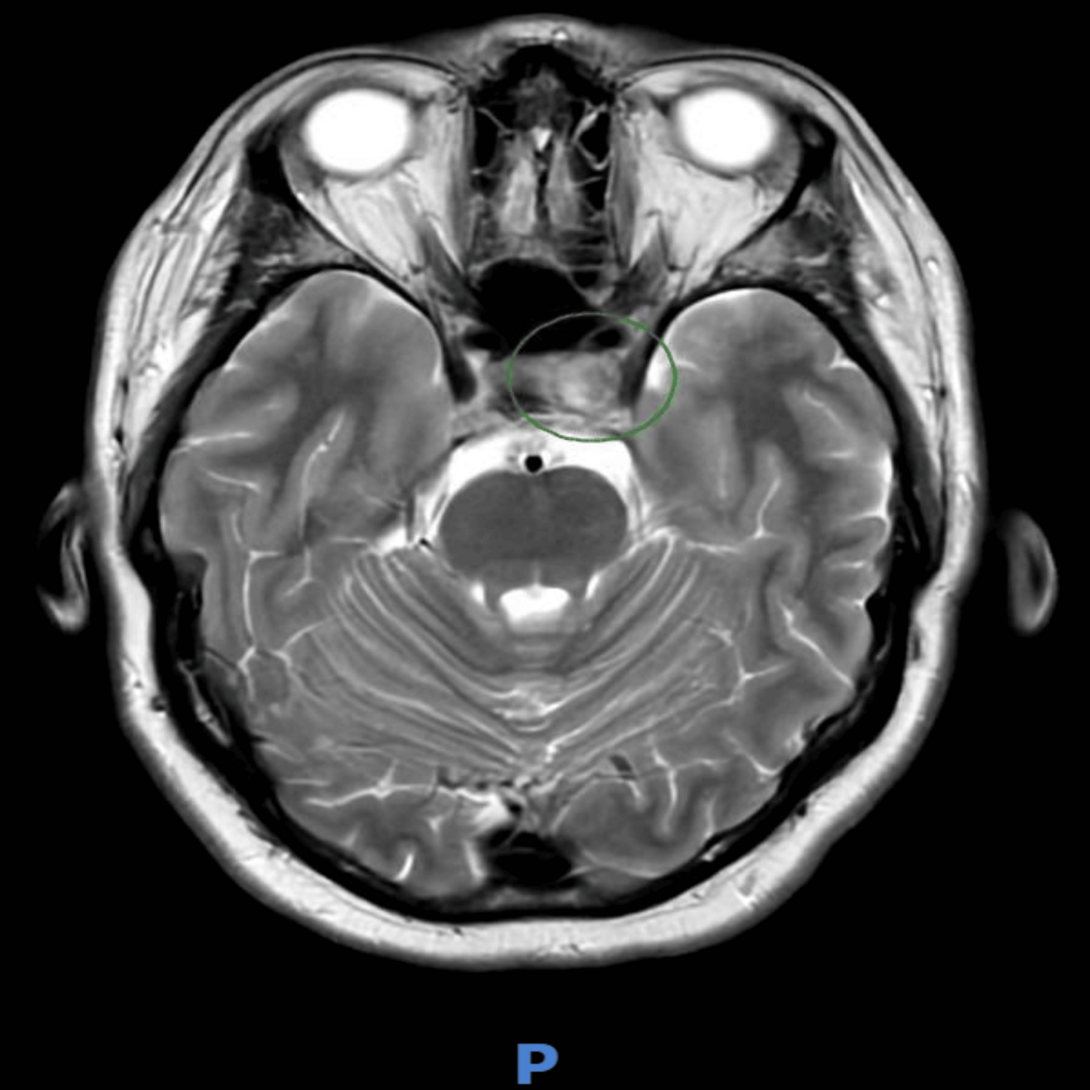
Pituitary MRI with contrast imaging. Asymmetrical enlargement of the left pituitary gland, which had a cranial-caudal thickness of 1 cm. Slight suprasellar extension, with midline pituitary stalk and no compression of the optic nerve/chiasm. On pre-contrast images, there was an abnormally high T1-weighted signal in the left lobe. On post-contrast images, there was a 1 x 1.2 x 1 cm corresponding area of reduced enhancement in the left loop, with a suggestion of partial encroachment onto the left cavernous sinus.

Although the CT of the head was unremarkable (Figure 2), MRI of the brain revealed a bulky left pituitary gland, predominantly affecting the left lobe (Figure 3). A subsequent MRI of the pituitary gland with IV contrast (Figure 4) demonstrated asymmetrical enlargement of the left pituitary gland with slight suprasellar extension and partial encroachment on the left cavernous sinus. In the pre-contrast images, there was an abnormally high T1-weighted signal in the left lobe. On post-contrast imaging, there was a 1 x 1.2 x 1 cm corresponding area of reduced enhancement in the left lobe. There was no evidence of optic nerve or chiasmal compression.

Routine laboratory investigations, including serum electrolytes, were within normal limits (results shown in Tables 2, 3). Capillary blood glucose (CBG) was also normal, ranging between 6.7 and 12.8 mmol/L (normal reference range: 4.0-14.0 mmol/L).

**Table 2:** Urea & electrolyte (U&E) results showing the comparison between the hospital's reference values and the patient's results.

Test	Normal range of values	Patient values
Sodium (Na)	133-146 mmol/L	139 mmol/L
Potassium (K)	3.5-5.3 mmol/L	4.1 mmol/L
Urea	2.5-7.8 mmol/L	3.5 mmol/L
Creatinine (Cr)	49-90 umol/L	61 umol/L

**Table 3:** Endocrine panel results showing the comparison between the hospital's reference values and the patient's results. * The hospital's laboratory range of values for peri-menopausal women.

Test	Normal range of values	Patient values
Cortisol	145-619 nmol/L	467 nmol/L
Testosterone	0.3-1.2 nmol/L	0.5 nmol/L
Prolactin	59-619 miu/L	219 miu/L
Thyroid-stimulating hormone	0.49-5.23 miu/L	0.42 miu/L
T4	11.5-22.7 pmol/L	15.1 pmol/L
T3	3.5-6.5 pmol/L	4.7 pmol/L
Insulin-like growth factor 1 (IGF-1)	6.0-24.0 nmol/L	28 nmol/L
Follicle-stimulating hormone	23-116 iu/L*	19.7 iu/L
Luteinizing hormone	7.9-53.8 iu/L*	11.6 iu/L
Estradiol	0-118.2 pmol/L*	130 pmol/L

Pituitary function tests demonstrated normal levels of cortisol, follicle-stimulating hormone (FSH), luteinizing hormone (LH), estradiol, and prolactin, supporting the diagnosis of a likely non-functioning pituitary macroadenoma. Thyroid-stimulating hormone (TSH) was slightly low at 0.42 miu/L, but with a normal T4 at 15.1 pmol/L, this possibly indicates a likely nonthyroidal illness. However, insulin-like growth factor 1 (IGF-1) was mildly elevated at 28.0. Despite these biochemical findings suggestive of a slight growth hormone dysfunction, the patient exhibited no clinical features of acromegaly, and there was no relevant family history. Hence, IGF-1 was likely mildly elevated due to laboratory or metabolic variation, as the patient had T2DM. At 54 years old, she was also biochemically peri-menopausal, which may have influenced certain elements of the pituitary hormone profile, particularly gonadotropin and estradiol levels (as denoted by the normal range of values marked with "*" in Table 3). An oral glucose tolerance test (OGTT) was therefore recommended to exclude excessive growth hormone secretion, alongside repeat thyroid function tests and dynamic assessment for possible hypocortisolism.

The team referred the patient to the local pituitary multidisciplinary team (MDT) for further evaluation.

Treatment

The patient’s initial management focused on symptomatic relief and endocrine support, with paracetamol 1,000 mg four times daily prescribed for analgesia.

The parent team treated the pituitary apoplexy with an initial intravenous dose of hydrocortisone 100 mg, followed by 50 mg four times daily for three days. They then tapered the regimen to oral hydrocortisone 15 mg daily to address potential hypocortisolism.

Given the imaging findings and involvement of the third cranial nerve, the team discussed the case with neurosurgery, who advised against immediate surgical intervention and recommended urgent endocrine and ophthalmology consultations.

Ophthalmologic assessment confirmed a partial surgical left third cranial nerve palsy with associated ptosis. However, there was noted improvement in ocular symptoms during admission with no intervention, so there was no indication for surgical or acute ophthalmologic intervention.

Outcome and follow-up

The patient was discharged home in a stable condition, with oral hydrocortisone and a structured follow-up plan. The parent team scheduled an ophthalmology review to repeat the visual field assessment, along with endocrinology follow-up to monitor pituitary function and detect any emerging hormonal abnormalities. They also arranged a follow-up review with the regional neurosurgical team at Walton Hospital following discussion at the pituitary MDT meeting. This coordinated approach ensures ongoing monitoring of her neurological status and pituitary function, supporting timely intervention if needed. Additionally, the patient’s IGF-1 levels are planned for re-testing in August, as the previous results during this hospital admission showed only a minimal elevation.

## Discussion

Pituitary apoplexy represents an acute expansion of intrasellar contents due to hemorrhage or infarction within a pituitary adenoma, leading to a rapid rise in intrasellar pressure. This pathophysiology explains the sudden and severe symptoms (including headache, cranial neuropathies, and visual disturbance) that characterize the condition. Although ophthalmoplegia most often occurs in the context of cavernous sinus extension, isolated surgical third cranial nerve palsy without chiasmal compression, as seen in this case, remains an uncommon presentation [5].

Current UK guidelines emphasize that management decisions should be based on the severity and progression of neuro-ophthalmic deficits. Surgical decompression is recommended for patients with reduced consciousness, progressive visual loss, or worsening cranial neuropathies; however, patients who are clinically stable or improving may be managed nonoperatively under close surveillance [5]. This framework supports the conservative approach taken in this case, given the absence of chiasmal involvement and the patient’s early improvement.

Growing evidence from recent multicenter and prospective studies reinforces that conservative management can be safe and effective in selected patients. Mamelak et al. demonstrated no significant difference in endocrine or visual outcomes between surgical and nonsurgical cohorts when patients were stratified by clinical severity rather than managed uniformly [6]. Abucham’s commentary further highlighted that while early surgery remains essential for severe presentations, stable patients can achieve excellent outcomes with medical therapy and structured follow-up [7].

The Spanish multicenter studies by Biagetti et al. and Cordero Asanza et al. provide additional support for individualized management. Lower pituitary apoplexy scores (the reference values of which are shown in Table 1), absence of chiasmal compression, older age, and non-functioning adenomas were associated with favorable outcomes under conservative therapy, including spontaneous radiological involution and resolution of cranial neuropathies [8,9]. These findings closely mirror the clinical trajectory of our patient, who experienced gradual improvement in ptosis and headache without evidence of visual field compromise.

Close endocrine and ophthalmologic follow-up remains crucial in conservatively managed cases to detect delayed hypopituitarism or interval tumor growth. This patient was referred to the neurosurgery and endocrine services for structured monitoring through the pituitary multidisciplinary team.

A limitation in this case is the absence of dynamic growth hormone axis testing, which may have clarified the significance of the mildly elevated IGF-1 level, although the overall clinical picture suggested a non-functioning adenoma with no biochemical evidence of acromegaly.

## Conclusions

This case underscores the importance of considering pituitary apoplexy as a differential diagnosis in patients presenting with acute, painful surgical third cranial nerve palsy and headache, even when visual fields are intact and radiologic mass effect is minimal. Early administration of corticosteroids remains essential to address potential adrenal insufficiency and limit inflammatory progression. In alignment with contemporary evidence and national guidelines, the absence of chiasmal compression, early neurological improvement, and overall clinical stability (and therefore, a low PAS) supported a conservative, nonoperative approach in this patient. Their progressive improvement in ptosis and headache, together with preserved visual function, reflects the favorable outcomes increasingly reported in selected cases managed without surgery. Ultimately, this case highlights the value of timely recognition, comprehensive hormonal assessment, and coordinated multidisciplinary follow-up. It reinforces that individualized, patient-centered decision-making (guided by clinical severity, imaging findings, and evolving evidence) remains central to achieving optimal outcomes in pituitary apoplexy.
